# Aspergillus fumigatus Acetate Utilization Impacts Virulence Traits and Pathogenicity

**DOI:** 10.1128/mBio.01682-21

**Published:** 2021-07-27

**Authors:** Laure Nicolas Annick Ries, Patricia Alves de Castro, Lilian Pereira Silva, Clara Valero, Thaila Fernanda dos Reis, Raquel Saborano, Iola F. Duarte, Gabriela Felix Persinoti, Jacob L. Steenwyk, Antonis Rokas, Fausto Almeida, Jonas Henrique Costa, Taicia Fill, Sarah Sze Wah Wong, Vishukumar Aimanianda, Fernando José Santos Rodrigues, Relber A. Gonçales, Cláudio Duarte-Oliveira, Agostinho Carvalho, Gustavo H. Goldman

**Affiliations:** a Faculdade de Medicina de Ribeirão Preto, Departamento de Bioquímica e Imunologia, Universidade de São Paulo, São Paulo, Brazil; b Faculdade de Ciências Farmacêuticas de Ribeirão Preto, Departamento de Ciências Farmacêuticas, Universidade de São Paulo, São Paulo, Brazil; c University of Birmingham, Institute of Cancer and Genomic Sciences, Birmingham, England; d CICECO - Aveiro Institute of Materials, Department of Chemistry, University of Aveiro, Aveiro, Portugal; e Brazilian Biorenewables National Laboratory (LNBR), Brazilian Center for Research in Energy and Materials (CNPEM), Campinas, Sao Paulo, Brazil; f Department of Biological Sciences, Vanderbilt University, Nashville, Tennessee, USA; g Instituto de Química, Departamento de Química Orgânica, Universidade de Campinas, Campinas, São Paulo, Brazil; h Molecular Mycology Unit, Institut Pasteur, CNRS, UMR2000, Paris, France; i Life and Health Sciences Research Institute (ICVS), School of Medicine, University of Minho, Braga, Portugal; j ICVS/3B’s - PT Government Associate Laboratory, Guimarães/Braga, Portugal; Karlsruhe Institute of Technology (KIT)

**Keywords:** *Aspergillus fumigatus*, acetate assimilation, cell wall, secondary metabolites, transcription factor

## Abstract

Aspergillus fumigatus is a major opportunistic fungal pathogen of immunocompromised and immunocompetent hosts. To successfully establish an infection, A. fumigatus needs to use host carbon sources, such as acetate, present in the body fluids and peripheral tissues. However, utilization of acetate as a carbon source by fungi in the context of infection has not been investigated. This work shows that acetate is metabolized via different pathways in A. fumigatus and that acetate utilization is under the regulatory control of a transcription factor (TF), FacB. A. fumigatus acetate utilization is subject to carbon catabolite repression (CCR), although this is only partially dependent on the TF and main regulator of CCR CreA. The available extracellular carbon source, in this case glucose and acetate, significantly affected A. fumigatus virulence traits such as secondary metabolite secretion and cell wall composition, with the latter having consequences for resistance to oxidative stress, antifungal drugs, and human neutrophil-mediated killing. Furthermore, deletion of *facB* significantly impaired the *in vivo* virulence of A. fumigatus in both insect and mammalian models of invasive aspergillosis. This is the first report on acetate utilization in A. fumigatus, and this work further highlights the importance of available host-specific carbon sources in shaping fungal virulence traits and subsequent disease outcome, and a potential target for the development of antifungal strategies.

## INTRODUCTION

Aspergillus fumigatus is a saprotrophic filamentous fungus and opportunistic pathogen of immunocompetent and immunocompromised hosts. Together with other opportunistic fungal pathogens, such as Candida albicans and Cryptococcus neoformans, globally they kill in excess of 1.5 million people a year (1). The severity of the diseases related to A. fumigatus depend on preexisting infections as well as on the status of the host immune system (2). To successfully colonize and survive within the human host, A. fumigatus needs to acquire and metabolize nutrients. Essential nutrients include minerals such as iron, copper, and zinc, which are required in small amounts, while carbon and nitrogen, the main energy sources for sustaining biosynthetic processes, must be obtained in large quantities (3). Iron, zinc, and copper acquisition and metabolism have been studied in A. fumigatus in the context of virulence (4–6), whereas less is known about carbon source acquisition and metabolism in this fungus during infection. Studies have inferred that glucose, lactate, and acetate are carbon sources available to fungi *in vivo* with their availability and concentration depending on the host niche (7, 8). Whereas glucose utilization has been shown to be important for A. fumigatus disease progression (9), the utilization of the physiologically relevant short-chain fatty acids (SCFAs) lactate and acetate remain unexplored in this fungus. Indeed, acetate was detected in bronchoalveolar lavage (BAL) fluid samples from healthy and immunosuppressed mice, suggesting the presence of this carbon source, independent of the underlying immune condition, at the A. fumigatus primary site of infection. Our work therefore aimed at characterizing A. fumigatus acetate utilization and its relevance for virulence.

In the human body, acetate is present in the blood plasma at concentrations ranging from 0.074 to 0.621 mM depending on the type of artery, diet, and alcohol intake (10). Peripheral tissues can consume acetate from the bloodstream and oxidize it (10). The main producer of plasma acetate is the gastrointestinal (GI) tract-resident microbiome, with the majority of GI-resident bacterial species being capable of producing acetate (10). Furthermore, acetate has immunoregulatory properties. Acetate is an agonist for the G-protein-coupled receptors (GPCRs) FFA2, FFA3, and GPR109A, which are expressed in a number of immune cells, thus affecting the production of cytokines, regulation of downstream anti- and proinflammatory responses, and recruitment of immune cells (11). Acetate is likely important during invasive fungal infections, as it can regulate immunity at distal sites, including the lungs.

As mentioned above, small quantities of acetate were detected in healthy and immunosuppressed mice (8). The lungs are lined with a mucosa and contain a microbiome that has been shown to suffer alterations in the presence of disease (12). The lung microbiome, just like the gut microbiome, may contribute to the production and secretion of SCFAs (13). Studies investigating the production of SCFAs and other molecules by the lung microbiota are nonexistent, probably due to the lungs having been thought of as sterile until a few years ago (12).

Our understanding of the utilization of potential food sources during infection mainly relies on *in vitro* transcriptional studies (14–16). Despite several studies having investigated A. fumigatus gene expression during *in vivo* infection of chemotherapeutic mouse models of invasive aspergillosis, none of these studies have characterized the modulation of genes encoding components required for carbon source utilization (17–20). The genome of A. fumigatus encodes the acetyl coenzyme A (acetyl-CoA) synthetases (ACS) FacA (Afu4g11080) and PcsA (Afu2g07780), with *facA* shown to be upregulated in conidia exposed to neutrophils, and the corresponding protein induced by heat shock and repressed during hypoxic conditions (14, 21, 22). In A. fumigatus, the homologue of the Aspergillus nidulans transcription factor (TF)-encoding *facB* gene is upregulated when conidia are exposed to neutrophils (14). Furthermore, A. fumigatus conidia which were exposed to human neutrophils from healthy or CGD (chronic granulomatous disease) donors showed an upregulation of genes encoding enzymes involved in the glyoxylate cycle, gluconeogenesis, peroxisome function, and fatty acid degradation (14), suggesting an induction of metabolic pathways that are required for the utilization of alternative, nonpreferred carbon sources. Together, the aforementioned studies suggest that the utilization of alternative carbon sources such as SCFAs is important for A. fumigatus infection.

Acetate utilization has been investigated in detail in the model fungus A. nidulans. In A. nidulans, acetate was shown to be transported by the short-chain carboxylate transporters AcpA and AcpB, with the former being expressed in germinating conidia and young germlings, and the latter expressed in mycelia (23). The genome of A. fumigatus encodes one homologue of both *acpA* and *acpB* (Afu2g04080), which remains uncharacterized. Once internalized, acetate is converted by ACS to acetyl-CoA, which subsequently is transported into the mitochondria where it enters the tricarboxylic acid (TCA) cycle for the synthesis of ATP molecules (24). Furthermore, acetate, in the acetyl-CoA form, is oxidized via the glyoxylate cycle and required during gluconeogenesis (24). In A. nidulans, acetate utilization is under the control of the TF FacB, which is transcriptionally induced in the presence of acetate (25). FacB controls the expression of the ACS FacA, carnitine acetyltransferases FacC, AcuH, and AcuJ, the succinate/fumarate antiporter AcuL, and the glyoxylate cycle malate synthase AcuE (25, 26). The genome of A. fumigatus encodes homologues of the A. nidulans components required for acetate metabolism, although they have not been investigated until now. This work characterized the utilization of acetate in A. fumigatus and highlights the importance of the type of available extracellular carbon source in shaping fungal virulence determinants.

## RESULTS

### Acetate is metabolized via the glyoxylate and TCA cycles and a precursor for different metabolites.

**FIG 1:**
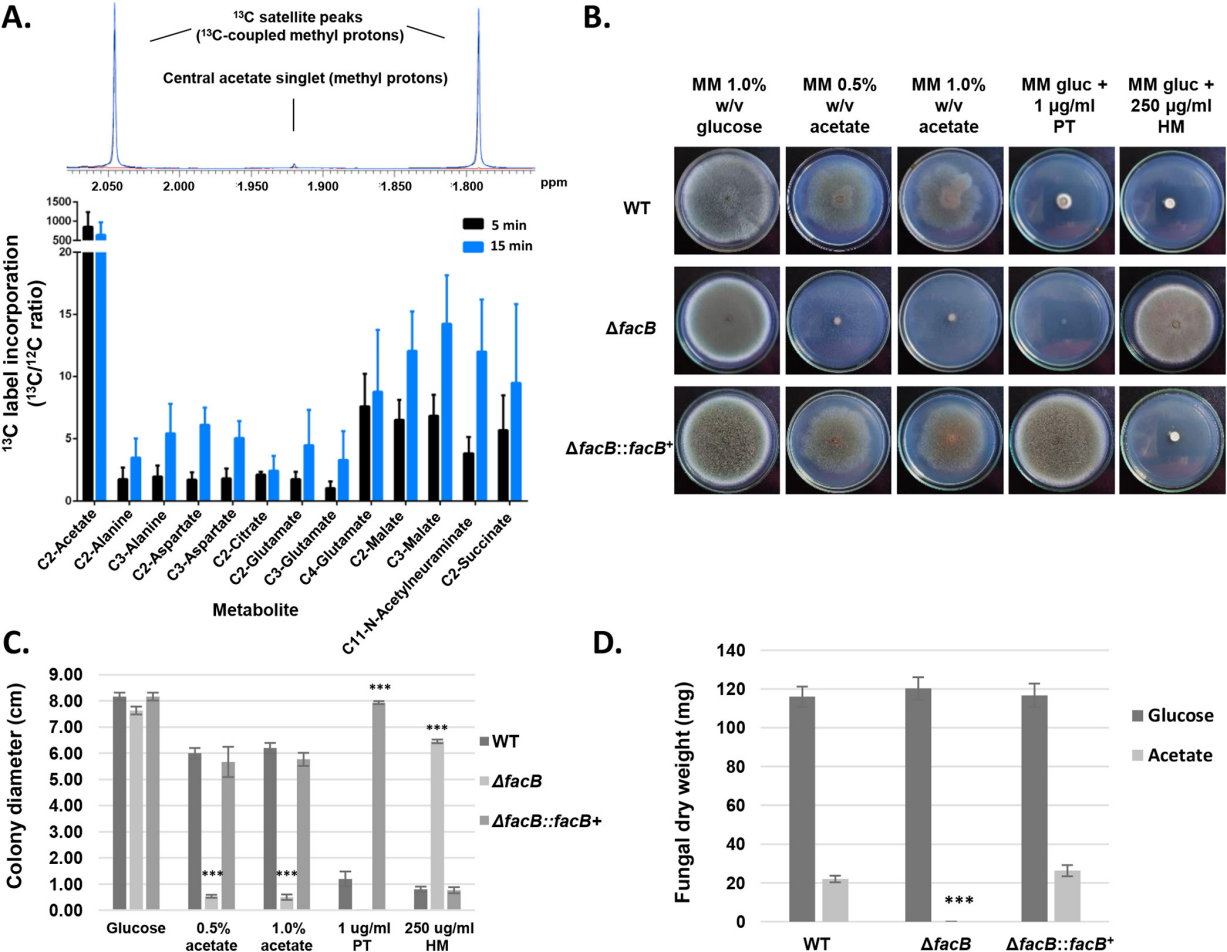
Acetate metabolism in A. fumigatus. (A) One- and two-dimensional (1D and 2D) NMR (nuclear magnetic resonance) analysis of ^13^C_2_-labeled acetate incorporation and metabolism. (Top) Expansion of 1D ^1^H NMR spectra of fungal cell extracts showing the increase in acetate carbon satellite peaks upon culture of A. fumigatus in ^13^C_2_-acetate-containing medium. (Bottom) ^13^C/^12^C ratios for metabolites that incorporated ^13^C derived from acetate, as determined through integration of ^1^H-^13^C HSQC (heteronuclear single quantum coherence) spectra recorded for extracts of fungal cells grown for 5 and 15 min in medium containing [^13^C_2_]acetate in comparison to nonlabeled control cultures. Values are averages plus standard deviations (error bars) of biological triplicates. (B to D) The transcription factor FacB is essential for growth in the presence of acetate. Strains were grown for 5 days (B and C) or 3 days (D) in either solid (B and C) or liquid (D) minimal medium (MM) supplemented with 1% (wt/vol) glucose (gluc) (B and D), 0.5% (wt/vol) (B) or 1% (wt/vol) (B and D) acetate before radial diameter (C) or fungal dry weight (D) was measured. To ensure homologous integration of *facB* in the complementation strain, strains were grown in the presence of pyrithiamine (PT) (*facB* was reintroduced into the Δ*facB* strain using the PT-resistant marker gene) and hygromycin (HM) (*facB* was deleted using the HM resistance marker gene) (B and C). Plate pictures (B) are representative for the average radial diameter shown in panel C. Values are averages ± standard deviations (error bars) of biological triplicates. The values for the *facB* deletion strain are significantly different from the values for the wild-type (WT) strain in a two-way multiple comparison analysis of variance (ANOVA) test as indicated: ***, *P* value of <0.0001.

To investigate acetate metabolism in A. fumigatus, the metabolic fate of acetate was traced by incubating fungal mycelia with ^13^C_2_-labeled acetate. The A. fumigatus wild-type (WT) strain CEA17 was grown for 16 h in peptone-rich minimal medium (MM) before undergoing 4 h of carbon starvation in MM. Subsequently, ^13^C_2_-labeled acetate was added to the cultures for 5 and 15 min before mycelia were separated from the culture medium and immediately snap-frozen in liquid nitrogen. Following metabolite extraction, one-dimensional (1D) ^1^H and two-dimensional (2D) ^1^H-^13^C HSQC (heteronuclear single quantum coherence) NMR (nuclear magnetic resonance) spectra were recorded for each sample. The uptake of ^13^C_2_-acetate by A. fumigatus was observed through significant increases in carbon satellite peaks (reflecting ^1^H-^13^C coupling) on both sides of the central acetate singlet (δ 1.92 ppm) in ^1^H spectra of fungal cell extracts (Fig. 1A, top). To identify metabolites that were directly derived from ^13^C_2_-labeled acetate and determine their fractional enrichment, ^1^H-^13^C HSQC spectra from 5- and 15-min samples were compared to spectra of control cells (4-h starvation, no labeling). The extent of ^13^C incorporation levels was obtained for each metabolite by dividing the 2D peak intensity in ^13^C-enriched samples by the peak intensity in the matched control sample (with 1.1% natural abundance in ^13^C levels). Results showed that labeled ^13^C from acetate was incorporated into the amino acids alanine at carbon-2 and carbon-3 (C2 and C3), aspartate at C2 and C3, and glutamate at C2, C3, and C4, the glyoxylate/tricarboxylic acid (TCA) cycle intermediates citrate at C2, malate at C2 and C3, and succinate at C2 as well as in the glycolipid and glycoprotein compound *N*-acetylneuraminate at C11 (Fig. 1A, bottom).

These results indicate that acetate is taken up and metabolized via the glyoxylate and TCA cycles in A. fumigatus, which is in agreement with studies of Saccharomyces cerevisiae (27) and A. nidulans (24) and in line with the metabolism of two carbon compounds as the sole carbon source. Indeed, ^13^C was incorporated into the amino acid and Krebs cycle intermediate aspartate (28) as well as the amino acids alanine and glutamate. The TCA cycle intermediate oxaloacetate is converted into phosphor-enol-pyruvate (gluconeogenesis), which in turn is a precursor for alanine in a two-step process that involves glutamate. Furthermore, ^13^C was also enriched in other cellular compounds such as *N*-acetylneuraminate, which is a sialic acid that is present on cell surface acidic glycoconjugates, and which has been shown to contribute to the phagocytic properties of cells of opportunistic fungal pathogens such as Cryptococcus neoformans (29), Candida albicans (30), and A. fumigatus (31). Hence, this analysis showed that acetate is metabolized by A. fumigatus through multiple pathways.

### The transcription factor FacB is essential for A. fumigatus growth in the presence of acetate and ethanol as the sole carbon sources.

To determine whether acetate utilization is controlled by a transcription factor (TF) in A. fumigatus, as was previously described for A. nidulans (25), a TF deletion library (32) was screened for reduced growth on plates containing MM supplemented with 0.5% (wt/vol) acetate (AMM) as the sole carbon source. Several strains were identified, and subsequent confirmation growth experiments, in both solid (radial growth) and liquid (dry weight) AMM, resulted in the selection of five strains that had reduced growth in acetate but presented no growth defects in glucose-rich MM (GMM) (Fig. 1B to D; see also Fig. S1A to C at https://doi.org/10.6084/m9.figshare.14740482). These strains were deleted for the *acuK* (Afu2g05830), *acuM* (Afu2g12330), *facB* (Afu1g13510), *farA* (Afu4g03960), and *mtfA* (Afu6g02690) genes (Fig. 1B to D; see also Fig. S1 at https://doi.org/10.6084/m9.figshare.14740482). AcuM, AcuK, and MtfA have been characterized in A. fumigatus and have been shown to be important for alternative carbon source utilization and virulence (33, 34). Furthermore, *farA* was shown to be important for fatty acid utilization and was upregulated in fungal cells exposed to human neutrophils (14). In contrast, A. fumigatus FacB, which is the homologue of A. nidulans FacB, remains uncharacterized. We therefore aimed at further deciphering the role of the TF FacB in A. fumigatus acetate utilization and virulence. FacB was also essential for growth in medium with ethanol as the sole carbon source but not for growth in the presence of different fatty acids (Fig. S1D at https://doi.org/10.6084/m9.figshare.14740482). Ethanol and acetate are two carbon compounds that require identical metabolic pathways with ethanol being converted to acetate via the metabolic intermediate acetaldehyde (35). Reintroduction of A. fumigatus *facB* in the Δ*facB* background strain at the *facB* locus through homologous recombination restored growth in acetate (Fig. 1B to D; see also Fig. S1D at https://doi.org/10.6084/m9.figshare.14740482), confirming that the FacB-encoding gene is essential for A. fumigatus growth on two carbon compounds.

### FacB controls acetate utilization through regulating genes encoding enzymes required for acetate metabolism.

To gain further insight into A. fumigatus acetate metabolism and to describe a role of FacB in the control of acetate utilization, the transcriptional response of the wild-type and Δ*facB* strains was assessed by RNA-sequencing (RNA-seq), when grown for 24 h in fructose-rich (control) MM and after transfer for 0.5 h (short incubation) or 6 h (long incubation) to MM supplemented with 0.1% (wt/vol) (low concentration) or 1% (wt/vol) (high concentration) acetate. We chose different concentrations of acetate and time points in order to decipher the transcriptional response in the presence of abundant and limiting carbon source concentrations after short and prolonged exposure. The number of significantly differentially expressed genes (DEGs) was defined as having a −1 < log_2_ fold change (log2FC) < 1 and an adjusted *P* value of <0.05 (Table 1; see also File S1 at https://doi.org/10.6084/m9.figshare.14740482). Two comparisons were carried out: (i) gene expression in the presence of the four different acetate conditions against gene expression in the control (fructose) condition in the WT strain and (ii) gene expression in the WT strain against gene expression in the Δ*facB* strain in the presence of the different acetate conditions (Table 1, eight comparisons in total).

**TABLE 1:** Number of differentially expressed genes (DEGs) (−1 < log2FC < 1) identified by RNA sequencing in the wild-type and Δ*facB* strains when grown for 0.5 h or 6 h in minimal medium supplemented with 0.1 or 1.0% (wt/vol) acetate

Comparison and condition	No. of upregulated genes (%)	No. of downregulated genes (%)	Total no. of genes
Comparing acetate vs fructose in the wild-type strain			
0.1% acetate 0.5 h	794 (54.7)	658 (45.3)	1,452
0.1% acetate 6.0 h	1,698 (54)	1,445 (46)	3,143
1% acetate 0.5 h	1,107 (47.8)	1,211 (52.2)	2,318
1% acetate 6.0 h	882 (66.3)	448 (33.7)	1,330
Comparing Δ*facB* vs wild-type strain			
0.1% acetate 0.5 h	34 (18.9)	145 (81.1)	179
0.1% acetate 6.0 h	710 (54.3)	596 (45.7)	1,306
1% acetate 0.5 h	54 (28.6)	134 (71.4)	188
1% acetate 6.0 h	482 (71.0)	198 (29)	678

Gene ontology (GO) and Functional Categorisation (FunCat) analyses could not be performed for many of the comparisons shown in Table 1, probably due to a low number of DEGs in some conditions (Table 1). DEGs were therefore manually inspected and divided into the following categories: (i) amino acid, protein, and nitrogen (urea, nitrate, ammonium) metabolism (degradation and biosynthesis); (ii) carbohydrate and lipid metabolism, including genes encoding enzymes required for lipid, fatty acid, and acetate degradation, CAZymes (carbohydrate active enzymes) and the metabolism of other sugars; (iii) cell signaling (protein kinases, phosphatases, regulators of G-protein signaling and G-protein-coupled receptors [GPCRs]); (iv) cell membrane and cell wall (ergosterol, chitin, and glucan biosynthesis/degradation); (v) miscellaneous (genes encoding enzymes with diverse functions that do not fit into the other categories); (vi) oxidation/reduction and respiration (oxidoreductases, monooxygenases, and respiratory chain enzymes); (vii) secondary metabolism; (viii) transcription factors; (ix) transporters (sugars, amino acids, ammonium, nitrate, ions, metals, and multidrug); (x) unknown (gene encoding proteins with unknown/uncharacterized functions); and (xi) putative virulence factors (proteases and proteins important for adhesion and interaction with the extracellular environment) (Files S2 and S3 at https://doi.org/10.6084/m9.figshare.14740482; Fig. S2 at https://doi.org/10.6084/m9.figshare.14740482). In the WT strain, this categorization was carried out for all DEGs with a −3 < log2FC < 3 in order to identify genes with the highest differential expression pattern. For comparisons between the WT and Δ*facB* strains, categorization was carried out for all DEGs with a −1.5 < log2FC < 1.5 in order to include as many DEGs as possible.

The majority of DEGs (34 to 46%) encoded proteins of unknown function, whereas genes encoding enzymes required for carbohydrate and carbon compound (cc) metabolism, oxidation/reduction and respiration, secondary metabolism, and transporters constituted 38 to 48% of all DEGs (Fig. S2 at https://doi.org/10.6084/m9.figshare.14740482), suggesting the presence of acetate influences the regulation of these processes. No particular enrichment for any of the aforementioned categories was found for the conditions studied here (Fig. S2 at https://doi.org/10.6084/m9.figshare.14740482). There were differences though in the type of secondary metabolites (SMs), transporters as well as respiratory and carbon source metabolism encoded by the DEGs (Fig. S2 at https://doi.org/10.6084/m9.figshare.14740482).

**FIG 2:**
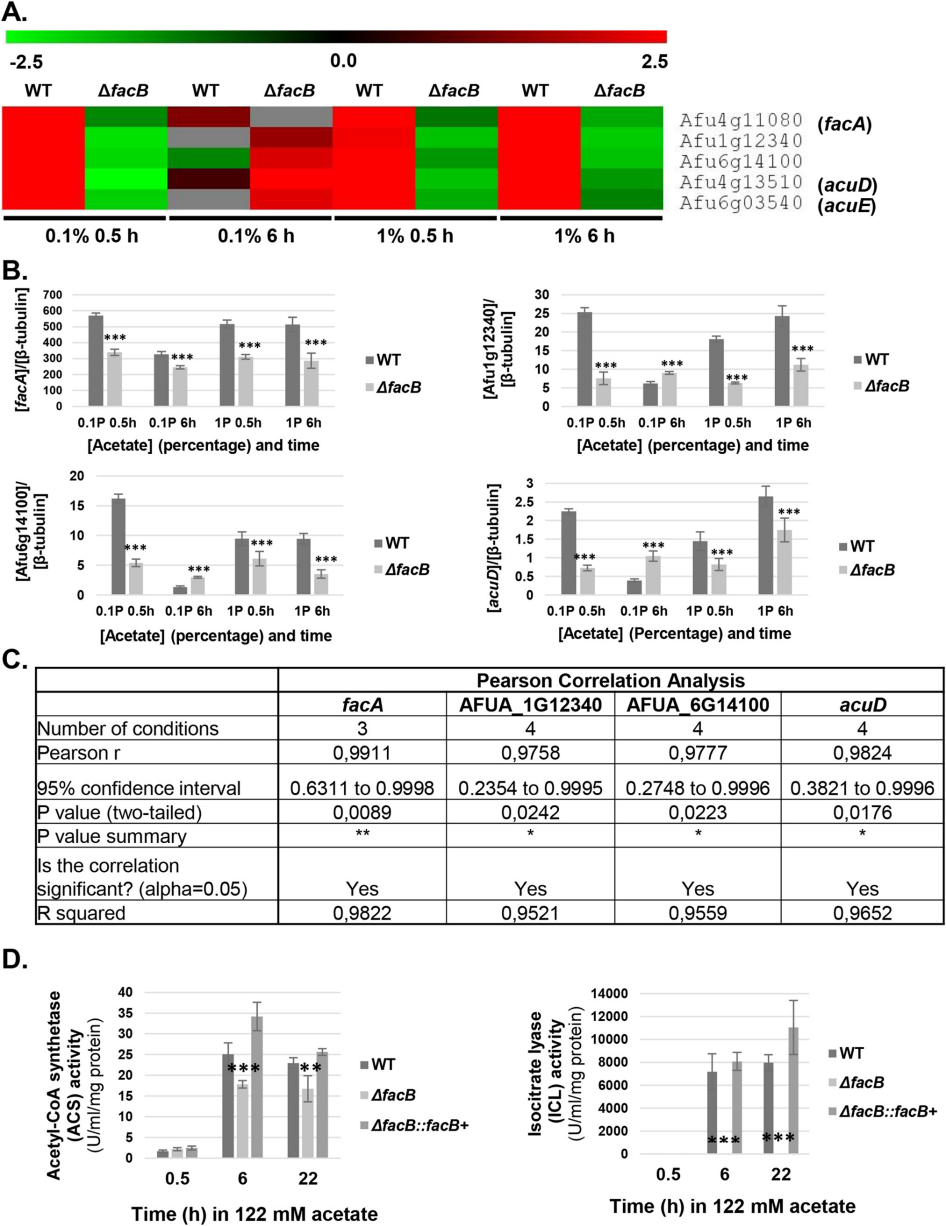
FacB regulates acetate metabolism. (A) Heat map depicting log2FC (log_2_ fold change) from the RNA-sequencing data of genes encoding enzymes required for acetate metabolism in the wild-type (WT) and Δ*facB* strains in the presence of 0.1% (wt/vol) or 1% (wt/vol) acetate after 0.5 h and 6 h. The log2FC for the WT strain is based on the comparison of gene expression between the WT strain grown in fructose-rich medium and after transfer to acetate-rich medium, whereas log2FC for the Δ*facB* strain is from the comparison between the WT and *facB* deletion strain for each acetate condition. (B) Validation of RNA-sequencing data by qRT-PCR shows that FacB is required for the transcriptional expression of genes encoding enzymes involved in acetate metabolism. Strains were first grown in minimal medium (MM) supplemented with fructose before mycelia were transferred to acetate-containing MM, RNA was extracted and reverse transcribed to cDNA, and qRT-PCR was run on genes *facA*, Afu1g12340, Afu6g14100, and *acuD*. Gene expression levels were normalized by β-tubulin. (C) Results of Pearson correlation analysis between the RNA-sequencing and qRT-PCR data sets for four genes encoding enzymes involved in acetate metabolism. Gene fold change values were used for the analysis, which was carried out in Prism GraphPad (*, *P* value < 0.05; **, *P* value < 0.005). (D) FacB is required for acetyl-CoA synthetase (ACS) and isocitrate lyase (ICL) activities. Strains were grown in fructose-rich MM for 24 h, before mycelia were transferred to MM containing 1% (wt/vol) acetate for 0.5 h, 6 h, and 22 h, total cellular proteins were extracted, and enzyme activities were measured. Values are averages ± standard deviations (error bars) of biological triplicates. The values for the *facB* deletion strain are significantly different from the values for the wild-type (WT) strain in a two-way multiple comparison analysis of variance (ANOVA) test as indicated: **, *P* value < 0.001; ***, *P* value < 0.0001.

To further unravel the role of FacB in acetate utilization, we focused on DEGs that encode enzymes important for acetate metabolism. In the wild-type strain, genes encoding the ACS FacA (but not the ACS PcsA), a carnitine acetyltransferase (Afu1g12340), a mitochondrial carnitine:acyl carnitine carrier (Afu6g14100), the isocitrate lyase (ICL) AcuD (*acuD*, glyoxylate cycle), and the malate synthase AcuE (*acuE*, glyoxylate cycle) were highly expressed under all acetate conditions; in contrast, these genes were repressed in the Δ*facB* strain (Fig. 2A). The exception was in the presence of 6 h in 0.1% (wt/vol) acetate, where these genes were not expressed in the WT strain but they were induced in the *facB* deletion strain (Fig. 2A). This is likely due to carbon starvation, which would occur under these conditions. Assessment of the expression of *facA*, Afu1g12340, Afu6g14100, and *acuD* by quantitative real-time reverse transcriptase (qRT-PCR) under the same conditions confirmed the RNA-seq data (Fig. 2B and C).

To further confirm the transcriptional data, we assayed the activities of ACS and ICL (isocitrate lyase) in the WT, Δ*facB*, and Δ*facB*::*facB*^+^ strains when grown in the presence of 1% (wt/vol) acetate for 0.5 h, 6 h, and 22 h. In agreement with the RNA-seq data, ACS and ICL activities were induced in the presence of acetate in the WT and Δ*facB*::*facB*^+^ strains. No significant difference in ACS and ICL activities were observed between the WT and Δ*facB*::*facB*^+^ strains, whereas these enzyme activities were significantly reduced in the Δ*facB* strain under all tested conditions (Fig. 2D). ICL activity was completely dependent on FacB with the loss of *facB* resulting in no enzyme activity (Fig. 2D). In contrast, ACS activity was reduced ∼20 to 30% in the Δ*facB* strain compared to the WT and Δ*facB*::*facB*^+^ strains (Fig. 2D). The observed ACS activity is likely due to the activity of the second A. fumigatus ACS PcsA. Our RNA-seq data show that *pcsA* is not under the regulatory control of FacB in the conditions tested here, whereas the expression of the single ICL-encoding gene *acuD*, is regulated by FacB (Fig. 2A). These results suggest that FacB controls acetate utilization through regulating genes encoding enzymes required for acetate metabolism.

### Acetate metabolism is subject to carbon catabolite repression.

**FIG 3:**
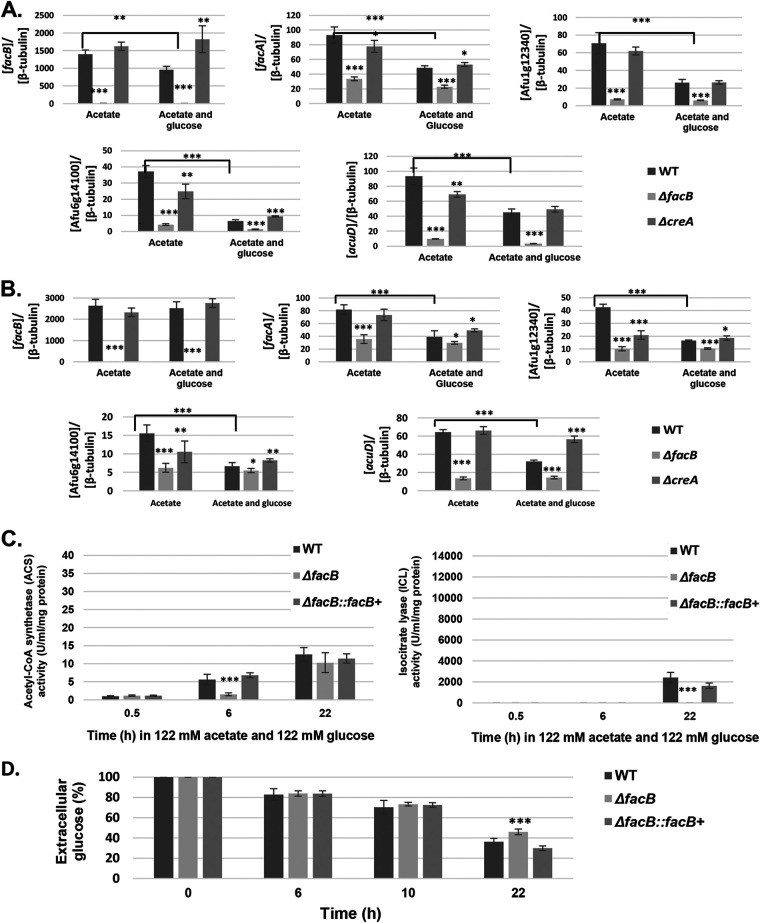
Acetate utilization is subject to carbon catabolite repression (CCR). (A and B) Expression of genes *facA*, Afu1g12340, Afu6g14100, and *acuD*, as determined by qRT-PCR, in strains grown for 24 h in minimal medium (MM) supplemented with fructose and then transferred for 0.5 h to MM supplemented with either acetate or acetate and glucose. The graphs in panel A show results from growth in 12.2 mM for each carbon source, whereas the graphs in panel B show results from growth in 122 mM for each carbon source. (C) Activities of acetyl-CoA synthetase (ACS) and isocitrate lyase (ICL) in strains incubated for 0.5 h, 6 h, and 22 h in MM supplemented with 122 mM acetate and 122 mM glucose. Strains were first grown for 24 h in fructose-containing MM before mycelia were transferred to acetate- and glucose-containing MM. (D) Percentage of residual glucose in supernatants of strains grown for 24 h in fructose-rich MM and after transfer to glucose-containing MM for a total time period of 22 h. Values are averages ± standard deviations (error bars) of biological triplicates. The values for the *facB* deletion strain are significantly different from the values for the wild-type (WT) strain in a two-way multiple comparison analysis of variance (ANOVA) test as indicated: *, *P* value < 0.01; **, *P* value < 0.001; ***, *P* value < 0.0001. The values for the WT strain under two conditions were compared (indicated by a line).

In A. fumigatus, carbon catabolite repression (CCR) is a cellular process which directs primary metabolism to the utilization of preferred carbon sources (glucose) and results in the repression of genes required for the utilization of alternative carbon sources (acetate) (36). The opportunistic yeast pathogen C. albicans is able to simultaneously use glucose and lactate, due to the loss of an ubiquitination site on ICL (37). This increased metabolic flexibility plays a role in the adaptation of C. albicans to the host environment with the addition of an ubiquitination site to C. albicans ICL resulting in decreased resistance to phagocytosis by macrophages, decreased fungal burden in the GI tract, and decreased dissemination to the kidneys (38). To determine whether A. fumigatus is able to use glucose and acetate simultaneously, transcriptional and enzymatic studies were performed in the WT and Δ*facB* strains in the presence of equimolar concentrations of glucose and acetate. We also included a strain deleted for the TF CreA, which is a transcriptional regulator of CCR (36). Strains were grown in the presence of 12.2 mM (0.1% [wt/vol]) and 122 mM (1% [wt/vol]) acetate without or with equimolar concentrations of glucose for 0.5 h, before expression of genes *facB*, *facA*, Afu1g12340, Afu6g14100, and *acuD* were determined by qRT-PCR (Fig. 3A and B). The presence of the different concentrations of glucose caused a significant downregulation of all genes, except for *facB* in the presence of 122 mM acetate and glucose (Fig. 3A and B). It is possible that the expression of *facB* is dependent on the concentration of the externally available carbon source. In Aspergillus spp., high- and low-affinity carbon source transporters are expressed depending on the concentration of the extracellular carbon source (39). A similar scenario can be envisaged for transcription factors (TFs), especially as they respond to external stimuli, with some TFs being highly induced under nutrient-limiting conditions and repressed in nutrient-sufficient conditions (20). Alternatively, Aspergillus transcriptions factors are not always regulated at the transcriptional level as previously shown (40). These results suggest that A. fumigatus acetate metabolism is subject to CCR as has been described in A. nidulans (25, 26).

In the presence of 12.2 mM acetate, deletion of *creA* caused a significant downregulation of *facA*, Afu6g14100, and *acuD*, whereas in the simultaneous presence of 12.2 mM acetate and glucose, the absence of *creA* significantly increased *facB* and Afu6g14100 gene expression (Fig. 3A). In the presence of 122 mM acetate, deletion of *creA* significantly reduced Afu1g12340 and Afu6g14100 gene expression, whereas in the presence of glucose, the expression of all genes, except for *facB*, was increased, although not to WT levels (Fig. 3B). The exception was the expression of *acuD* in the Δ*creA* strain in the presence of 122 mM acetate and glucose, which was similar to the expression levels of *acuD* in the WT strain in the presence of 122 mM acetate (Fig. 3B). These results suggest that (i) CreA may be involved in the control of genes required for alternative carbon source utilization and that (ii) acetate metabolism (with the exception of *acuD*) is partially dependent on CreA-mediated CCR in a concentration-dependent manner.

Next ACS and ICL activities were measured in the presence of 122 mM acetate and glucose after 0.5 h, 6 h, and 22 h. Enzyme activities were lower in the presence of acetate and glucose (Fig. 3C) than in the presence of acetate only (Fig. 2D), supporting the observed transcriptional repression of the corresponding genes in the presence of glucose. Basal ACS activity was detected under all conditions (likely due to the presence of the FacB-dependent ACS FacB and the FacB-independent ACS PcsA), whereas ICL activity was not detected at 0.5 h and 6 h. This is in agreement with the transcriptional data, suggesting that ICL activity is completely dependent on FacB for induction (Fig. 2B and C and Fig. 3B and C) and CreA for repression (Fig. 3B). After 22 h of incubation in both carbon sources, enzyme activities increased, which may be due to low glucose concentrations (∼30%) in the culture medium (Fig. 3D), making acetate the predominant available carbon source. Furthermore, significantly more extracellular glucose was present in supernatants of the Δ*facB* strain (Fig. 3D), suggesting that FacB may also be involved in the utilization of other carbon sources. Together, these results suggest that genes and enzymes involved in acetate utilization are subject to CCR and that their regulation is partially controlled by CreA.

### The extracellular carbon source influences the levels of secreted secondary metabolites.

**FIG 4:**
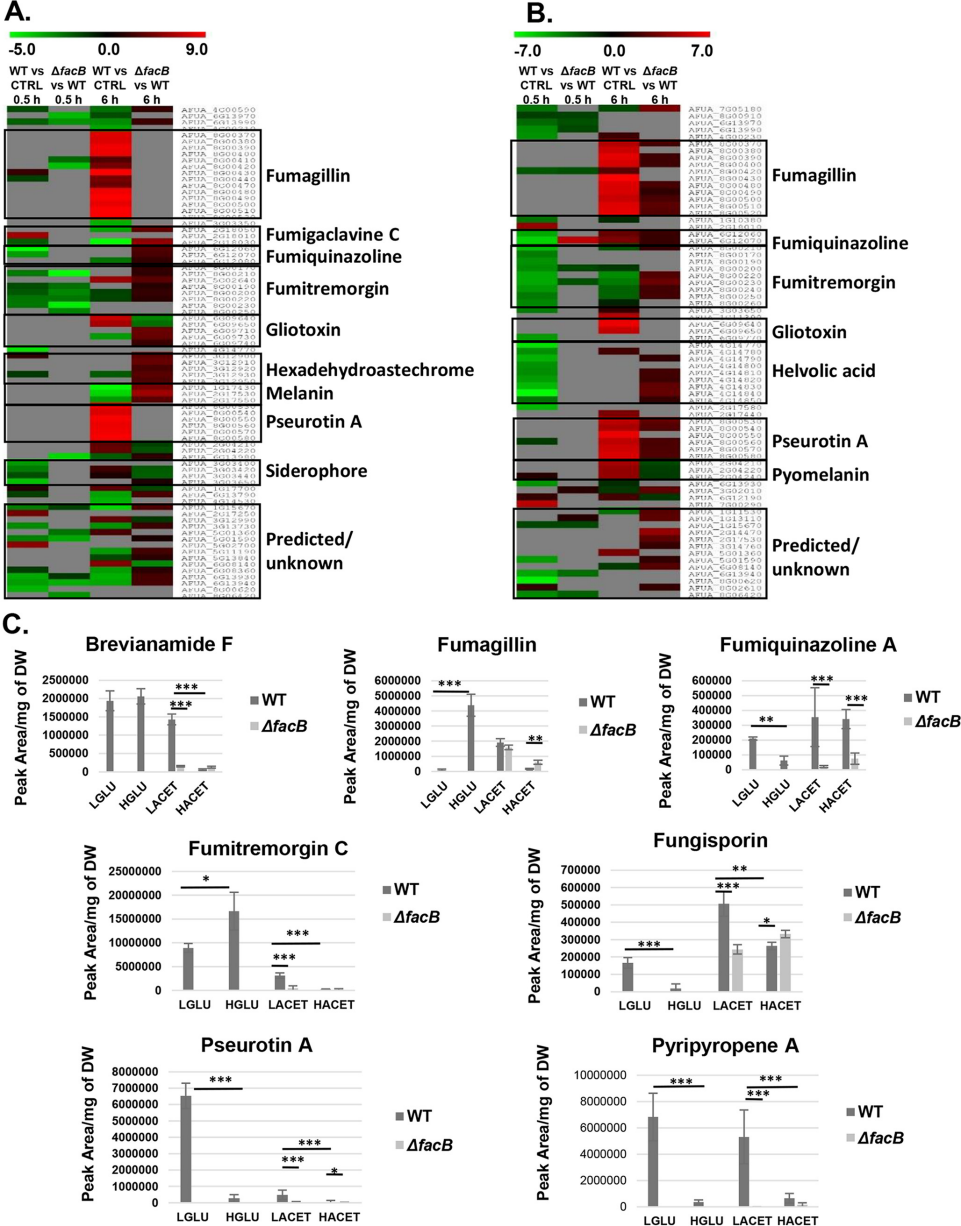
The extracellular carbon source affects the levels of secreted secondary metabolites (SMs). (A and B) Heat map of the log_2_ fold change (FC), as determined by RNA-seq, of genes predicted to encode enzymes required for SM biosynthesis in the wild-type (WT) and Δ*facB* strains when grown for 0.5 h and 6 h in the presence of 0.1% (wt/vol) or 1% (wt/vol) acetate or when comparing gene expression in the WT strain in the presence of different acetate concentrations and in the presence of fructose (control [CTRL] condition). Genes that did not show a significant FC are shown in gray. (C) Quantities of identified SMs, as determined by high-performance liquid chromatography (HPLC), in the WT and Δ*facB* strains when grown for 24 h in minimal medium supplemented with 0.1% (wt/vol) (LGLU, low glucose; LACET, low acetate) or 1% (wt/vol) glucose (HGLU, high glucose; HACET, high acetate) or acetate. SM quantities were normalized by fungal dry weight (DW). Values are averages ± standard deviations (error bars) of four biological replicates. Values that are significantly different in a two-way multiple comparison analysis of variance (ANOVA) test are indicated as follows: *, *P* value < 0.01; **, *P* value < 0.001; ***, *P* value < 0.0001.

The secretion of secondary metabolites (SMs) has been shown to be essential for A. fumigatus proliferation within the natural environment and mammalian host, for evasion and modulation of the host immune system, and for virulence (41). Our RNA-seq data show that many DEGs are part of the fumagillin, pseurotin A, pyomelanin, and gliotoxin SM biosynthetic gene clusters (BGCs) (Fig. 4A and B; see also Fig. S2 at https://doi.org/10.6084/m9.figshare.14740482). SM BGCs are mainly expressed in the WT strain in the presence of 1% (wt/vol) acetate or after 6 h of incubation in MM supplemented with 0.1% (wt/vol) acetate (Fig. 4A and B). In contrast, these DEGs have reduced expression or are repressed in the Δ*facB* strain (Fig. 4A and B). Furthermore, the expression profiles of these genes were often reversed between the WT and Δ*facB* strains under these conditions, suggesting that the metabolic pathways regulated by FacB are important for SM gene expression.

To determine whether SMs are secreted specifically in the presence of acetate and dependent on FacB, high-performance liquid chromatography (HPLC) was performed on culture supernatants from the WT and Δ*facB* strains grown for 24 h in fructose-rich MM and after transfer to MM supplemented with 0.1% (wt/vol) and 1% (wt/vol) acetate for 24 h. This pregrowth in fructose ensured that the starting biomass was similar for all samples. In addition, SM profiles were examined for the WT strain when grown under the same conditions, with the exception that acetate was replaced with glucose as the main carbon source. After 24 h, a total of 18 SMs, including the previously characterized (42, 43) fumiquinazolines A and D, fumitremorgin C, pyripyropene A, pseurotins A and F2, fungisporin, and brevianamide F, were identified in culture supernatants from strains grown under all conditions (Table S2 at https://doi.org/10.6084/m9.figshare.14740482). We did not detect gliotoxin or pyomelanin in culture supernatants.

Subsequently, the concentrations of characterized SMs were quantified to determine whether the extracellular available carbon source influences the levels of secreted SMs. In the WT strain, concentrations of all these SMs, with the exception of fumiquinazoline and fungisporin, were significantly higher in the presence of 1% (wt/vol) glucose than in the presence of 1% (wt/vol) acetate (Fig. 4C). Similarly, in the presence of 0.1% (wt/vol) glucose, secreted levels of brevianamide F, fumiquinazoline, fumitremorgin C, and pseurotin A were significantly higher compared to concentrations in the presence of 0.1% (wt/vol) acetate, whereas levels of fumagillin and fungisporin A were significantly higher in the presence of 0.1% (wt/vol) acetate than in the presence of 0.1% glucose (Fig. 4C). Furthermore, differences in the levels of secreted SMs were also seen between the two different concentrations of the same carbon source (Fig. 4C). In addition, deletion of *facB* resulted in a significant decrease in the concentrations of secreted SMs in the presence of different concentrations of extracellular acetate with the exception of fumagillin (Fig. 4C).

Together, these results suggest that the concentration and type of available extracellular carbon source affect the levels of secreted SMs.

### The composition of the A. fumigatus cell wall is carbon source dependent.

In A. fumigatus, the composition of the culture medium influenced cell wall composition, thus modulating their sensitivity to antifungal agents (44). In addition, primary carbon metabolism was shown to influence cell wall content and/or organization (45). To investigate whether glucose and acetate, representing preferred and alternative carbon sources, respectively, would also influence cell wall composition, we determined the quantities of cell wall polysaccharides in the A. fumigatus WT strain when grown in the presence of each of these carbon sources.

**FIG 5:**
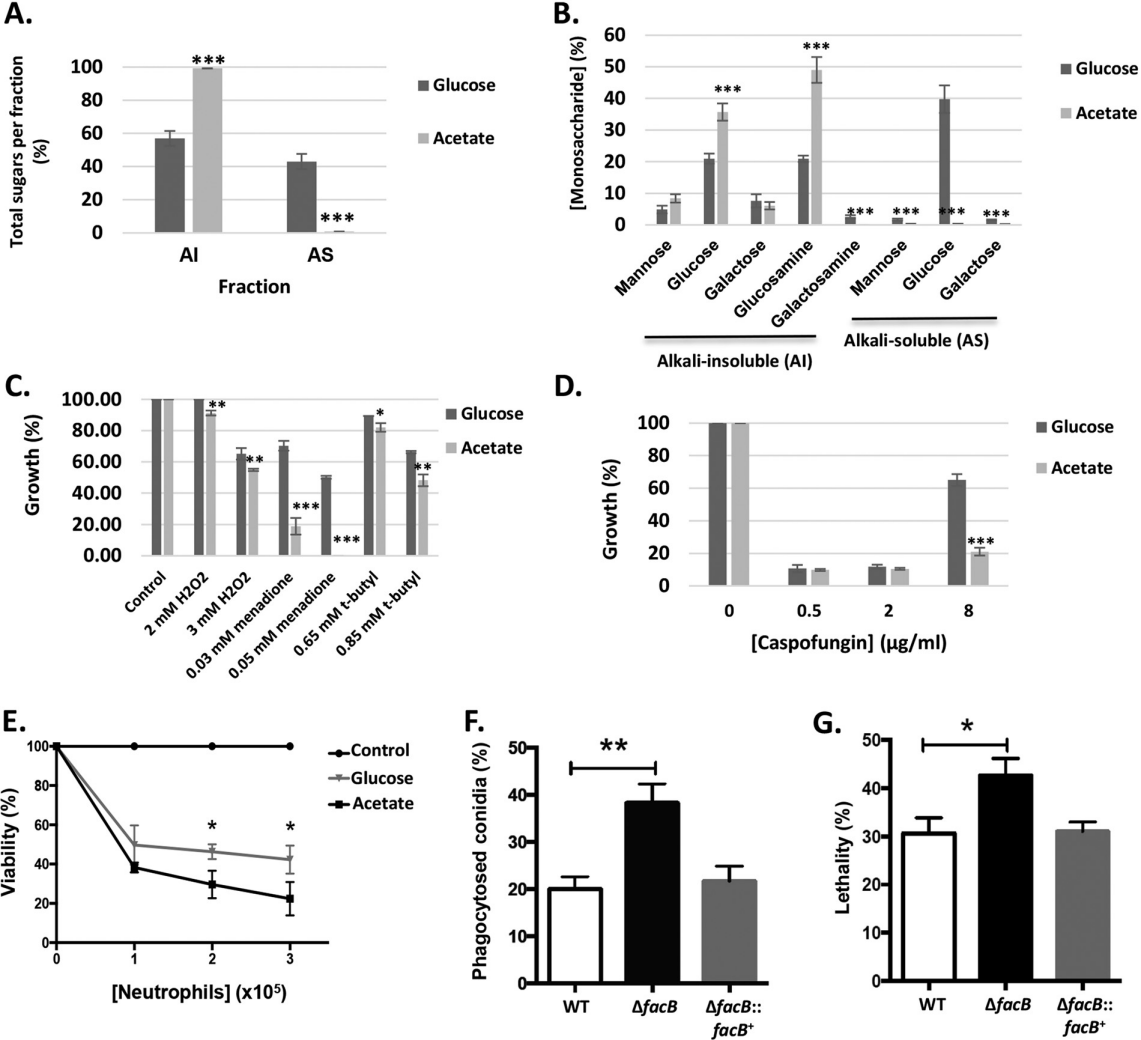
Acetate utilization impacts cell wall polysaccharide content, oxidative stress, caspofungin, and immune cell resistance in A. fumigatus. (A and B) Percentage of total (A) and individual (B) sugars identified in the alkali-insoluble (AI) and alkali-soluble (AS) fractions by gas liquid chromatography of the WT strain when grown for 24 h in MM supplemented with glucose and acetate. Values are averages ± standard deviations (error bars) of four biological replicates. The values for the acetate condition are significantly different from the values for the glucose condition in a two-way multiple comparison analysis of variance (ANOVA) test as indicated: ***, *P* value < 0.0001. (C) The wild-type (WT) strain was grown from 10^5^ spores for 5 days on minimal medium containing glucose (GMM) or acetate (AMM) supplemented with different concentrations of oxidative stress-inducing compounds. Colony diameters were measured and normalized by the control condition and expressed as percentage of growth in comparison to the control condition. (D) As described above for panel C, with the exception that GMM or AMM was supplemented with increasing concentrations of caspofungin. Values are averages ± standard deviations (error bars) of three biological replicates. Values that are significantly different for the acetate condition compared to the value for the glucose condition in a two-way multiple comparison analysis of variance (ANOVA) test are indicated as follows: **, *P* value < 0.001; ***, *P* value < 0.0001. (E) The WT strain was pregrown for 8 h or 13 h in GMM or AMM, respectively, before hyphae were incubated with different concentrations of human neutrophils for 1 h. Subsequently, cells were lysed, and hyphal viability was assessed via an MTT assay and calculated. Values are averages ± standard deviations (error bars) of three biological replicates. Values that are significantly different for the acetate condition compared to the value for the glucose condition in a one-tailed *t* test are indicated as follows: *, *P* value < 0.05. (F and G) Murine bone marrow-derived macrophage (BMDM) phagocytosis (F) and killing (G) of A. fumigatus conidia. BMDMs were incubated with fungal conidia before they were stained with calcofluor white, and the percentage of phagocytosed conidia was assessed by microscopy and calculated. To assess fungal viability, conidium-macrophage mixtures were lysed, diluted, and inoculated on plates containing complete medium before CFU were assessed and the percentage of viability was calculated. Values are averages ± standard deviations (error bars) of three biological replicates. Values that are significantly different in a paired *t* test are indicated as follows: *, *P* value < 0.05; **, *P* value < 0.005.

Cell wall alkali-insoluble (AI) and alkali-soluble (AS) fractions were prepared from WT mycelia grown for 24 h in MM supplemented with 1% (wt/vol) glucose or acetate and analyzed by gas-liquid chromatography (Fig. S3A at https://doi.org/10.6084/m9.figshare.14740482). Results show that there is a significant increase in the percentage of the cell wall AI fraction in the presence of acetate due to increased concentrations of glucose (β-1,3-glucan) and glucosamine (chitin) (Fig. 5A and B; Fig. S3A at https://doi.org/10.6084/m9.figshare.14740482). In contrast, the percentage of cell wall AS fraction was significantly reduced in the presence of acetate predominantly due to decreased levels of glucose (α-1,3-glucan), although significantly decreased levels of mannose and galactose were also observed (Fig. 5A and B; Fig. S3A at https://doi.org/10.6084/m9.figshare.14740482). These results suggest that the type of extracellular carbon source significantly influences A. fumigatus cell wall composition and organization.

### Oxidative stress and antifungal drug tolerance are carbon source dependent.

The A. fumigatus cell wall has been shown to play a major role during infection as it represents the main line of defense for the fungus and is responsible for interacting with and modulating host immune cells as well as for oxidative stress and antifungal drug resistance (46). The aforementioned results show that the type of carbon source has an effect on cell wall polysaccharide concentrations. Subsequently, oxidative stress and antifungal drug resistance were determined in the A. fumigatus WT strain when grown in the presence of glucose or acetate.

First, the WT strain was grown in the presence of GMM or AMM supplemented with the oxidative stress-inducing compounds hydrogen peroxide (H_2_O_2_), menadione, and *t*-butyl hydroperoxide, before colony diameters were measured and the percentage of growth was normalized by the growth in the control, drug-free condition for each carbon source. Growth was significantly reduced in the presence of AMM supplemented with the oxidative stress-inducing compounds compared to growth in the presence of GMM supplemented with the oxidative stress-inducing compounds (Fig. 5C; see also Fig. S3B at https://doi.org/10.6084/m9.figshare.14740482). These results suggest that the presence of acetate increases sensitivity to oxidative stress in A. fumigatus.

Next, we determined resistance to antifungal drugs, including different azoles, amphotericin B and caspofungin, when the WT strain was grown in the presence of glucose and acetate. We performed MIC assays of amphotericin B, voriconazole, itraconazole, and posaconazole when A. fumigatus was grown in RPMI 1640 medium (standard reference medium used for MIC assays), GMM, and AMM for 72 h. A. fumigatus grown in the presence of AMM was slightly more susceptible to amphotericin B than compared to growth in the presence of RPMI 1640 and GMM (Table 2). No difference in susceptibility was observed for the different azoles tested here compared to RPMI 1640, although reduced growth in the presence of these azoles was observed when comparing MIC between GMM and AMM (Table 2). The WT strain was also grown in GMM or AMM supplemented with increasing concentrations of the echinocandin and second line therapy drug caspofungin (47). In the presence of 0.5 and 2 μg/ml caspofungin, growth was severely inhibited and did not differ between both carbon sources (Fig. 5D; see also Fig. S3C at https://doi.org/10.6084/m9.figshare.14740482). At 8 μg/ml caspofungin, the WT strain had increased growth in the presence of glucose and acetate, due to the caspofungin paradoxical effect (increased fungal growth in the presence of higher caspofungin concentrations [48]). In the presence of acetate, the WT was less able to recover growth compared to growth on GMM (Fig. 5D; see also Fig. S3C at https://doi.org/10.6084/m9.figshare.14740482).

**TABLE 2:** MICs of different antifungal drugs on the A. fumigatus WT strain grown in different media^*a*^

Medium	MIC (μg/ml)^*b*^			
	Amphotericin B	Voriconazole	Itraconazole	Posaconazole
RPMI	3.33 ± 1.15	0.25 ± 0.00	0.42 ± 0.14	0.67 ± 0.29
GMM	3.33 ± 1.15	0.33 ± 0.14	0.50 ± 0.00	1.00 ± 0.00
AMM	1.17* ± 0.76	0.21 ± 0.07	0.33 ± 0.14	0.67 ± 0.29

a RPMI 1640 medium (RPMI) containing glucose (GMM) or acetate (AMM).

b Values are averages ± standard deviations of three independent repeats. The value for AMM is significantly different from the values for RPMI and GMM (*P* < 0.05 by a two-way ANOVA test) as indicated by the asterisk.

These results suggest that oxidative stress and antifungal drug resistance change depending on the extracellular, available carbon source.

### Acetate-grown hyphae are more susceptible to human neutrophil-mediated killing than hyphae grown in the presence of glucose.

The aforementioned results indicate that growth in the presence of acetate influences virulence determinants such as SM production, cell wall composition, oxidative stress, and antifungal drug resistance compared to growth in the presence of energetically more favorable carbon sources such as glucose. To determine the role of carbon source-mediated growth for resistance against human neutrophils, we assayed the viability of hyphae, pregrown in MM supplemented with either glucose or acetate as the sole carbon source. To ensure that a similar number of conidia had germinated prior to incubation with neutrophils, microscopy was performed, and the number of germinated conidia was counted. After incubation for 8 h in GMM and 13 h in AMM, ∼90% of conidia had germinated under both conditions (Fig. S3D at https://doi.org/10.6084/m9.figshare.14740482), and they were visually inspected to be similar in length (data not shown). Human neutrophils used at different multiplicities of infection (MOIs), killed significantly more (60 to 80%) A. fumigatus hyphae grown in AMM than compared to hyphae (50 to 60%) pregrown in GMM (Fig. 5E). These results indicate that hyphae grown in acetate-rich medium are more susceptible to human neutrophil-mediated killing than hyphae grown in the presence of glucose.

### FacB is crucial for virulence in insect and murine models of disseminated and invasive pulmonary aspergillosis.

Last, we assessed the virulence of the Δ*facB* strain *in vitro* and *in vivo*. First, the capacity of murine bone marrow-derived macrophages (BMDMs) to phagocytose and kill WT, Δ*facB*, and Δ*facB::facB*^+^ conidia was determined. The Δ*facB* strain was significantly more susceptible to BMDM phagocytosis (Fig. 5F), and a significantly larger number of Δ*facB* conidia were killed in comparison to the WT and Δ*facB::facB*^+^ strains (Fig. 5G).

**FIG 6:**
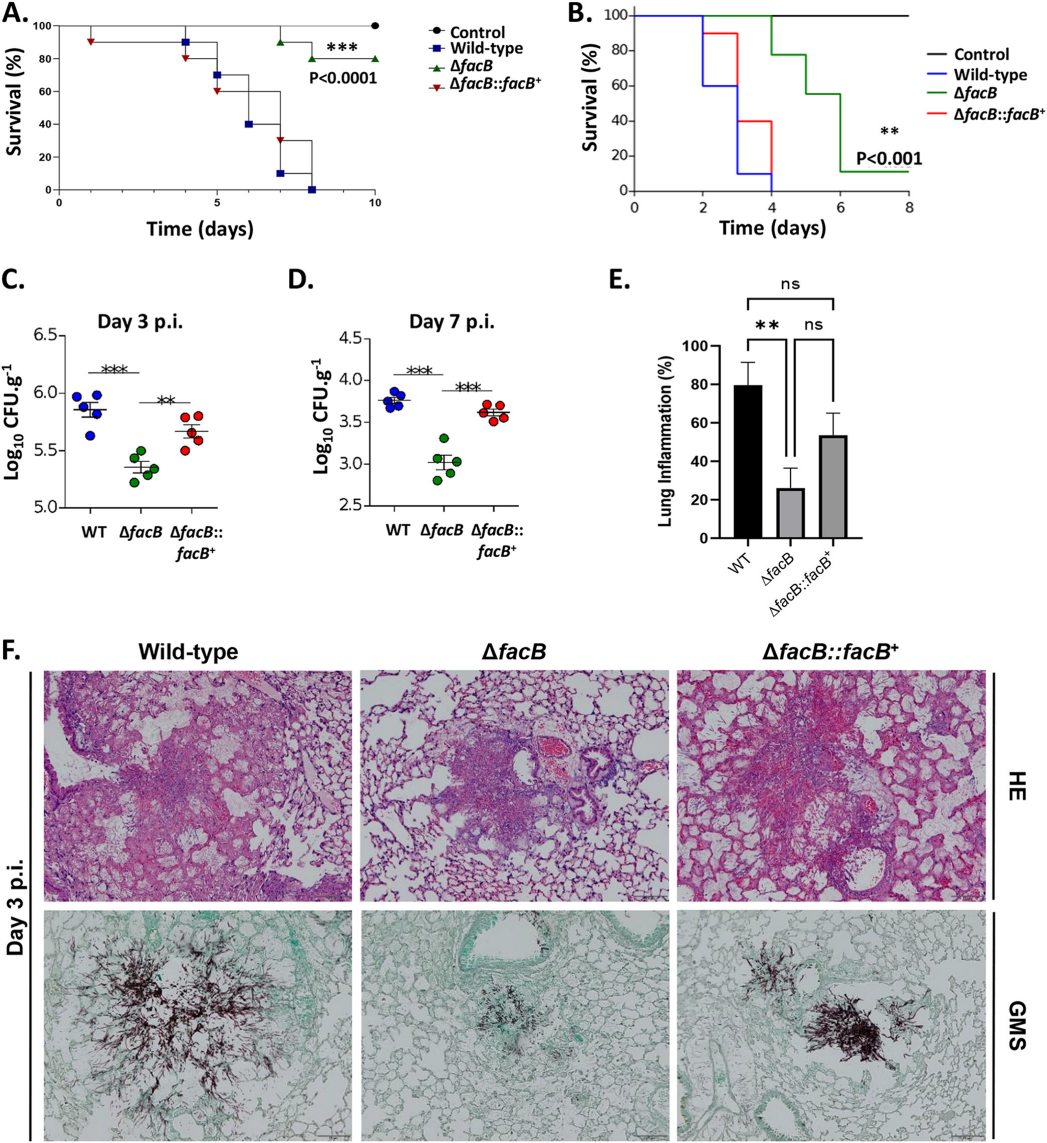
FacB is crucial for virulence in both insect and murine models of invasive aspergillosis. (A and B) Survival curves (*n* = 10/strain and *n* = 5 for control) of Galleria mellonella (A) and mice (B) infected with the respective A. fumigatus strains. Phosphate-buffered saline (PBS) without conidia was given as a negative control. The indicated *P* values are based on the log rank, Mantel-Cox, and Gehan-Breslow-Wilcoxon tests comparing the *facB* deletion strain to the WT and *facB* complemented strains. (C and D) Fungal burden in murine lungs after 3 (C) and 7 (D) days postinfection (p.i.) with the different A. fumigatus strains. Murine lungs were excised, ruptured, and resuspended, before dilutions were prepared that were incubated on plates containing complete medium. Fungal growth was assessed by counting the CFU on the plates for each dilution. (E) Inflammation in murine lungs after 3 days postinfection (p.i.) with the different A. fumigatus strains. Murine lungs were excised, and slides of lung sections were prepared. To quantify lung inflammation of infected animals, inflamed areas on slide images were analyzed using the thresholding tool in ImageJ software. Values are averages ± standard deviations (error bars) of three biological replicates (lungs from different mice). Values that are significantly different in a two-way multiple comparison ANOVA test are indicated as follows: *, *P* value < 0.01; **, *P* value < 0.001; ***, *P* value < 0.0001. (G) Histopathology of mice infected with the different A. fumigatus strains. Lungs were excised at 3 days postinfection (p.i.) before lung sections were prepared and stained with HE (hematoxylin and eosin) or with Grocott’s methenamine silver (GMS).

Next, virulence of the WT, Δ*facB*, and Δ*facB*::*facB*^+^ strains was determined in the wax moth Galleria mellonella and in a neutropenic murine model of invasive pulmonary aspergillosis (IPA). We used different animal models as virulent A. fumigatus was shown to be dependent on the status of the host immune system (49). In G. mellonella (Fig. 6A) and in chemotherapeutic mice (Fig. 6B), the Δ*facB* strain was hypovirulent compared to the WT and Δ*facB*::*facB*^+^ strains. In the insect model, the WT and Δ*facB*::*facB*^+^ strains killed all larvae after 8 days, whereas 80% of larvae infected with the Δ*facB* strain survived after 10 days (Fig. 6A). Similarly, the WT and Δ*facB*::*facB*^+^ strains killed all mice after 4 days, whereas 10% of mice infected with the Δ*facB* strain survived 6 days postinfection (p.i.) (Fig. 6B). In agreement, fungal burden was significantly reduced for the Δ*facB* strain after 3 (Fig. 6C) and 7 (Fig. 6D) days p.i. in murine lungs compared to the WT and Δ*facB*::*facB*^+^ strains. In addition, histopathology analyses of murine lungs after 3 p.i. showed significantly reduced inflammation (Fig. 6E and F) and growth in the lungs (Fig. 6F) for the Δ*facB* strain after 3 days p.i. compared to the WT and Δ*facB*::*facB*^+^ strains. Together, these results suggest that FacB is important for A. fumigatus virulence in insect and mammalian hosts.

To determine whether the observed reduction in virulence of the *facB* deletion strain may be due to growth defects, the WT, Δ*facB*, and Δ*facB*::*facB*^+^ strains were grown for 72 h in the presence of different media that are similar to the mammalian host environment before fungal biomass was quantified. The Δ*facB* strain had significantly reduced growth in the presence of DMEM (Dulbecco’s modified Eagle’s medium) containing glucose (low and high levels of glucose), FBS (fetal bovine serum), and beef extract compared to the WT and Δ*facB::facB*^+^ strains but not in the presence of RPMI 1640 medium and minimal medium supplemented with glucose (control) (Fig. S3E at https://doi.org/10.6084/m9.figshare.14740482). These results suggest that the observed reduction in virulence of the Δ*facB* strain is at least partially due to strain-specific growth defects in a mammalian host environment.

## DISCUSSION

This work aimed at deciphering the regulation of the physiologically relevant carbon source acetate in A. fumigatus and at determining its relevance for fungal virulence. As a first step, we show that A. fumigatus can take up and metabolize acetate via the TCA and glyoxylate cycles which is in agreement with studies of S. cerevisiae and A. nidulans (24, 27). Furthermore, A. fumigatus acetate metabolism was shown to be under the regulatory control of the transcription factor FacB, which controls the expression of genes encoding enzymes that are required for the conversion of acetate to acetyl-CoA, for mitochondrial import of acetyl-CoA and enzymes of the glyoxylate cycle. In agreement with the transcriptional data, deletion of *facB* also affected ACS (conversion of acetate to acetyl-CoA) and ICL (glyoxylate cycle) enzyme activities. These results are in agreement with studies of A. nidulans, where utilization of acetate as the sole carbon source is also dependent on FacB, with this TF regulating the expression of the ACS-encoding gene *facA* and the glyoxylate cycle enzyme-encoding genes *acuD* (ICL) and *acuF* (malate synthase) (25).

In addition, we show that A. fumigatus acetate metabolism-related genes as well as ACS and ICL activities are subject to CCR. This is in contrast to findings in C. albicans where the addition of glucose to lactate-grown cells did not result in CCR (38). It is important to note here that the experimental conditions differed between our study and the study in reference 38 (e.g., A. fumigatus growth in acetate and glucose versus C. albicans growth in lactate and then the addition of glucose). It is well-known that the addition of glucose to cultures causes CCR in Aspergillus spp. (36), and the rationale here was therefore to present the fungus with equimolar concentrations of both carbon sources during all growth stages. Acetate metabolism was also reported to be subject to CCR in A. nidulans, with *facB* and the carnitine acetyltransferase-encoding gene *facC* being under the regulatory control of the CC repressor CreA (50, 51).

In A. fumigatus, CreA-dependent repression of genes encoding enzymes required for acetate metabolism was observed only for the ICL-encoding gene *acuD*, whereas the other genes were only partially dependent on CreA-mediated repression. A discrepancy between *acuD* transcript levels and protein activity was observed. It is possible that basal transcript levels are present under all conditions to allow the fungus to quickly respond to changes in extracellular available nutrient sources, but that posttranscriptional processing is not taking place. Indeed, gene transcript levels cannot predict protein levels and activity due to mRNA spatiotemporal fluctuations and availability of protein synthesis components (52). Our data suggest that additional repressor proteins and/or mechanisms exist. In agreement, in A. nidulans, CreA has been shown to be part of a protein complex that mediates target gene repression and that corepressor proteins are crucial for CreA function (53). Furthermore, deletion of A. fumigatus *creA* resulted in significantly decreased expression of acetate utilization genes in the presence of acetate, suggesting that CreA may be involved in the regulation of these genes in the absence of glucose. In A. nidulans, CreA was shown to be important for growth in different carbon, nitrogen, and lipid sources and for amino acid metabolism (40), whereas in the filamentous fungus Trichoderma reesei, CRE1 was proposed to have roles in chromatin remodeling and developmental processes and was shown to also act as a transcriptional activator (54). These studies suggest that CreA and its homologues have additional roles, other than mitigating CCR, in filamentous fungi.

The deletion of *facB* caused a significantly differential expression of genes that are part of SM biosynthetic gene clusters (BGCs) as well as in secreted SMs. A direct correlation between transcript and secreted protein levels is not possible, as gene transcript levels cannot predict concentrations of biosynthesized proteins, due to intrinsic fluctuations in mRNA and availability of protein synthesis components (52). Furthermore, SM BGC regulation is extremely complex and governed by many TFs and epigenetic modifications, which result in the expression of different SM BGCs in any given condition (55). Subsequently, not all of these SMs are secreted, and transcriptional expression of gliotoxin and pyomelanin BGCs, as observed here, may be a consequence of the biosynthesis and secretion of other SMs. The role of FacB in the regulation of SM biosynthesis is perhaps not surprising, as this TF regulates genes encoding enzymes of central carbon metabolic pathways (e.g., glyoxylate cycle, shown in this work) during growth on alternative carbon sources. SMs are known to be derived from these central metabolic pathways (55), and this study further emphasizes that the amount of SMs produced occurs in a carbon source-dependent manner. Alternatively, the observed low concentrations of secreted SMs of the Δ*facB* strains may be due to the inability of this strain to grow in the presence of acetate. Notably, SMs measured in the conditions defined here may be secreted in response to carbon starvation, especially in the presence of low (0.1%) glucose and acetate conditions. These results further emphasize the role of FacB in regulating carbon metabolism. In addition, these results suggest that the concentration of secreted SMs *in vivo* is likely to also depend on host carbon sources and on carbon source starvation, which is encountered within different, nutrient-poor host niches.

Utilization of different carbon sources also affects the composition of the A. fumigatus cell wall, a factor crucial for fungal virulence, pathogenicity, and survival within the human host (2). This study shows that growth on acetate results in increased concentrations of the structural polysaccharides β-1,3-glucan and chitin and reduced levels of the cementing, “glue-like” α-1,3-glucan compared to the A. fumigatus cell wall after growth in glucose. Changes in cell wall composition are likely due to differences in primary carbon metabolism that govern the utilization of these carbon sources and that generate the cell wall polysaccharide precursors (45). Indeed, impairments in A. fumigatus glucose utilization metabolic pathways resulted in an altered cell wall (45). In agreement with other studies, our work suggests that these changes in cell wall composition influence A. fumigatus susceptibility to physiologically relevant stresses and antifungal drugs (44, 56). Likely, the significant reduction in the cementing α-1,3-glucan disturbs the organization of the other cell wall polysaccharides, increasing fungal cell wall permeability and susceptibility to extracellular stresses. This is true for the β-1,3-glucan synthase inhibitor caspofungin and for amphotericin B, which physiochemically interacts with membrane sterols (57). In contrast, increased susceptibility of acetate-grown hyphae to azoles, a class of antifungal drugs that impair ergosterol biosynthesis through targeting lanosterol demethylase of the ergosterol biosynthetic pathway, was not observed. A possible explanation for this may be that caspofungin and amphotericin B both target cell wall and cell membrane components, whereas azoles target intracellular enzymes and that despite the differences in cell wall organization and polysaccharide content, azole uptake is not affected. In agreement with our data where growth on acetate increases sensitivity to oxidative stress-inducing compounds, A. fumigatus acetate-germinated hyphae were more susceptible to human neutrophil-mediated killing *in vitro* compared to hyphae grown in the presence of glucose. The observed increase in susceptibility to different immune cells is probably due to the differences in cell wall composition resulting from growth in both carbon sources. Our observations are in agreement with a previous study, which showed that a hypoxic environment influenced cell wall thickness, composition, and surface-exposed polysaccharides, subsequently increasing neutrophil and macrophage reactiveness and activity against A. fumigatus (58). The physiological significance of the aforementioned acetate-related differences in cell wall composition, antifungal drug and oxidative stress resistance, and interaction with neutrophils remains to be determined. Although acetate was shown to present in the BAL fluid of mice (8), we currently do not know how acetate concentrations fluctuate within certain parts of the lung and the surrounding tissues, as has previously been shown for lung hypoxic microenvironments. It will be interesting to study the distribution of carbon sources in different host niches in future studies.

The Δ*facB* strain exhibited increased susceptibility to macrophage-mediated phagocytosis and killing. Due to the inability of the Δ*facB* strain to grow in the presence of acetate, we were unable to quantify cell wall composition in this strain and therefore determine whether this is a contributing factor when challenged with BMDMs. The inability of the Δ*facB* strain to use acetate may account for the observed increase in phagocytosis and killing of this strain, especially as acetate is available as a carbon source in macrophages (59). In agreement, the expression of genes required for acetate utilization in the presence of macrophages has been observed for A. fumigatus and prokaryotic pathogens (14, 60). It is unlikely that the observed increased phagocytosis and killing of this strain is due to defects in the glyoxylate cycle, as previous studies have revealed that glyoxylate cycle enzymes are dispensable for A. fumigatus virulence (61, 62). This is further supported by our findings that the utilization of fatty acids, which results in acetyl-CoA production via β-oxidation (26), is independent of FacB.

Furthermore, the Δ*facB* strain was hypovirulent in both insect and murine models of invasive aspergillosis. Reduced growth of the *facB* deletion strain in media simulating the host environment may account for the reduction in virulence observed for this strain. In addition to acetate metabolism, other FacB-controlled metabolic pathways, which are required for growth in these highly complex nutrient sources may be important for pathogenicity. Investigating the virulence of strains deleted for genes encoding components involved in central metabolic pathways such as the phosphoenolpyruvate (PEP) carboxykinase AcuF, the ACS FacA, and acetyl-CoA mitochondrial and peroxisome import proteins is subject to future investigations and may further explain the observed reduction in growth. Additional mechanisms may exist, which are important for virulence and are regulated by FacB, especially as the FacB regulon is large. Our RNA-seq data show that FacB regulates genes encoding proteins important for the production of SMs and oxidoreduction processes, which contribute to virulence. In addition, we cannot rule out that FacB has different targets *in vitro* compared to *in vivo*, as was previously shown for the A. fumigatus AcuM and AcuK transcription factors that are involved in the regulation of gluconeogenesis and iron acquisition (33). The exact mechanism of FacB for *in vivo* virulence thus remains to be determined but is possibly a combination of the aforementioned factors.

In summary, this study describes acetate utilization in A. fumigatus and highlights the importance of carbon source utilization and metabolic pathways for determining a variety of fungal traits that are crucial for virulence and that potentially shape disease outcome. Future studies should focus on this neglected area of exploring carbon source variety and availability in host primary sites of infection in order to better understand fungal pathogen nutrient requirements and utilization, which can potentially be targeted for developing antifungal strategies.

## MATERIALS AND METHODS

### Strains and media.

All strains used in this study are listed in Table S1 at https://doi.org/10.6084/m9.figshare.14740482. Reintroduction of *facB* through homologous combination at the *facB* locus was carried out by cotransformation of the Δ*facB* background strain with the *facB* (amplified by PCR) open reading frame (ORF) (no promoter) and the pyrithiamine-containing plasmid pPTR I at a ratio of 2:1. Homologous reintegration of *facB* in the Δ*facB* locus was confirmed by PCR and by growth assays (Fig. 1B). The *facB* deletion mutant was constructed using hygromycin as a selectable marker (32) and is therefore resistant to hygromycin and susceptible to pyrithiamine (Fig. 1B). Homologous reintegration of *facB* at the *facB* locus will result in the loss of the hygromycin gene. As pyrithiamine (PT) was used as a marker gene for construction of the reintegration mutant, the resulting strain is resistant to PT (Fig. 1B). Growth medium composition was exactly as described previously (40). Radial growth was determined after 5 days, whereas dry weight was measured after 3 days of growth. All growth was performed at 37°C, and experiments were performed in biological triplicates. Reagents were obtained from Sigma unless otherwise specified.

### Nuclear magnetic resonance analysis.

Metabolites were extracted from 5 mg freeze-dried fungal mycelia and dried in a speed vacuum as described previously (28). Extracts were reconstituted in 50 μl of deuterated sodium phosphate buffer (100 mM, pH 7.0) containing 0.5 mM trimethylsilylpropanoic acid (TMSP), 3 mM sodium azide, and 100% D_2_O. Each sample was sonicated for 10 min and vortexed briefly, before a volume of 35 μl was transferred into 1.7-mm nuclear magnetic resonance (NMR) tubes.

Spectra were acquired on a Bruker 600 MHz spectrometer equipped with a TCI 1.7-mm z-PFG cryogenic probe and a Bruker SampleJet autosampler. One-dimensional (1D) ^1^H NMR spectra and 2D ^1^H-^13^C HSQC (heteronuclear single quantum coherence) spectra were recorded and analyzed for each sample as previously described (63).

### RNA extraction and cDNA biosynthesis.

RNA was extracted with TRIzol (Invitrogen) as described previously (40), and 1 μg of RNA was reverse transcribed to cDNA using the ImPromII reverse transcriptase kit (Promega), according to the manufacturer’s instructions.

### RNA sequencing.

The quality of the RNA was assessed using the Agilent Bioanalyser 2100 (Agilent Technologies) with a minimum RNA integrity number (RIN) value of 7.0. Illumina sequencing was used for sample RNA sequencing as described previously (64). Libraries were prepared using the TruSeq Stranded mRNA LT Set B kit (Illumina) and sequenced (2 × 100 bp) on the LNBR NGS sequencing facility HiSeq 2500 instrument. RNA sequencing (RNA-seq) data were processed (quality check, cleanup, and removal of rRNA and genome mapping) as described previously (64) with the following modifications. The Bioconductor package tximport (version 1.12.3) was used to import raw read counts into DESeq2 (version 1.24.0), which subsequently quantified differential gene expression. The default Benjamini and Hochberg method was used for multiple hypothesis correction of DESeq2 differentially expressed genes.

### Enzyme activities.

Total cellular proteins were extracted as described previously (28), and isocitrate lyase (ICL) activity was measured and calculated as described previously (28). Acetyl-CoA synthetase (ACS) activity was measured and calculated as described previously (65), with the exception that intracellular proteins were extracted as described above and ACS activity was determined in 50 μg total intracellular protein.

### High performance liquid chromatography coupled to tandem mass spectrometry and data analysis.

Fungal biomass was separated from supernatant by miracloth before 20-ml portions of culture supernatants were freeze-dried. Secondary metabolites (SMs) were extracted from 100-mg freeze-dried samples by resuspending them in 1 ml high-performance liquid chromatography (HPLC)-grade methanol and sonicating them for 1 h in an ultrasonic bath. Samples were filtered and dried under a nitrogen stream before being resuspended in 1 ml of HPLC-grade methanol. Next, 100-μl samples were diluted in 900 μl of methanol and passed through 0.22-μm filters into vials.

HPLC coupled to tandem mass spectrometry (MS/MS) analysis was performed using a Thermo Scientific QExactive Hybrid Quadrupole-Orbitrap mass spectrometer. Parameters were as follows: positive mode, +3.5 kV capillary voltage; 250°C capillary temperature; 50 V S-lens and an *m/z* range of 133.40 to 2,000.00. MS/MS was performed using a normalized collision energy (NCE) of 30 eV, and five precursors per cycle were selected. For the stationary phase, the Thermo Scientific Accucore C_18_ 2.6-μm column (2.1 mm × 100 mm) was used. The mobile phase was carried out using 0.1% formic acid (A) and acetonitrile (B), and the following gradient was applied: 0 to 10 min, 5% B up to 98% B; hold for 5 min; 15 to 16.2 min, 95% B up to 5% B; hold for 8.8 min. The total run time was 25 min, and the flow rate was 0.2 ml min^−1^ with 3-μl injection volume. Data analysis was conducted using the Xcalibur software, version 3.0.63 (Thermo Fisher Scientific).

Molecular networks were made using the Global Natural Products Social Molecular Networking (GNPS) website (https://ccms-ucsd.github.io/GNPSDocumentation/ from http://gnps.ucsd.edu). First, all MS/MS fragment ions within 17 Da of the precursor *m/z* were removed, and spectra were filtered by choosing only the top six fragment ions in the ±50-Da window for the entire spectrum. The precursor ion mass tolerance and the MS/MS fragment ion tolerance were set at 0.02 Da. Subsequently, networks were created where edges were filtered to have a cosine score higher than 0.6 and more than five matched peaks. Edges between two nodes were kept in the network only if each of the nodes appeared in each other’s respective top 10 most similar nodes. Finally, the maximum size of a molecular family was set at 100, and the lowest scoring edges were removed. Network spectra were then searched against the GNPS spectral libraries, and library spectra were filtered in the same manner as the input data. Matches between network spectra and library spectra were filtered to have a score higher than 0.6 and at least five matching peaks (66). GNPS data used in this work are available at https://gnps.ucsd.edu/ProteoSAFe/status.jsp?task=f815e5618b05433fb768299a351fb793 (72-h data).

### Cell wall polysaccharide quantification.

Strains were grown for 24 h from 1 × 10^8^ conidia in 50 ml minimal medium (MM) supplemented with 1% (wt/vol) glucose or sodium acetate. Mycelia were harvested by vacuum filtration, washed, resuspended in 30 ml of double-distilled water (ddH_2_O), and disrupted using 5 ml of 0.5-mm glass beads in the FastPrep (MP Biomedicals) homogenizer at 4°C with two cycles of 60 s (6.0 vibration unit) and a 5-min interval between the two cycles. Samples were centrifuged at 5,000 rpm for 10 min at 4°C, before the cell wall-containing pellets were washed three times with ddH_2_O, resuspended in 15 ml of 50 mM Tris-HCl (pH 7.5), 50 mM EDTA, 2% (wt/vol) sodium dodecyl sulfate (SDS), and 40 mM β-mercaptoethanol, and boiled twice for 15 min in a water bath. Samples were centrifuged at 5,000 rpm for 10 min and washed five times with ddH_2_O. Resultant cell wall fractions were freeze-dried, and the dry weight was measured. Alkali fractionation of the cell wall was carried out by incubating the fractions twice in 1 M NaOH containing 0.5 M NaBH_4_ at 70°C for 1 h. Samples were centrifuged to separate supernatant (alkali-soluble [AS] fraction) from the pellet (alkali-insoluble [AI] fraction). The AI fractions was washed six times with ddH_2_O and centrifuged at 5,000 rpm for 10 min and freeze-dried. The excess of NaBH_4_ in the alkali-soluble fraction was neutralized with 2% (vol/vol) acetic acid, dialyzed against water until they achieved a neutral pH, and freeze-dried. Subsequently, AI and AS fractions were subjected to gas liquid chromatography as previously described (67).

### MICs.

MICs of amphotericin B and azoles on the A. fumigatus wild-type (WT) strain were carried out as described previously (68) with the exception that the WT strain was also grown in MM supplemented with glucose (GMM) or acetate (AMM).

### Neutrophil-mediated killing of hyphae.

Assessing the viability of A. fumigatus hyphae in the presence of human neutrophils was carried out as described previously with modifications (69). Briefly, human polymorphonuclear cells (PMNs) were isolated from 8 ml of peripheral blood from adult male healthy volunteers by density centrifugation and resuspended in Hanks balanced salt solution (Gibco). A. fumigatus conidia (1 × 10^8^) were incubated for 8 h or 13 h at 37°C in 30 ml GMM or AMM on a rotary shaker before they were centrifuged for 5 min at 4,000 rpm, supernatants were discarded, and pellets were resuspended in 1 ml PBS (phosphate-buffered saline). To assess the percentage of germinated conidia, samples were viewed under a microscope (Zeiss) at ×100 magnification before a total of 100 conidia were counted and the percentage of germinated conidia was calculated. Pregrown hyphae were then incubated with neutrophils (0, 1, 2 or 3 × 10^5^ cells/ml) for 1 h at 37°C in RPMI 1640 medium before cells were lysed and the MTT [3-(4,5-dimethylthiazol-2-yl)-2,5-diphenyltetrazolium bromide] assay was performed. Hyphal viability was calculated as a percentage of its viability after incubation without neutrophils.

### Bone marrow-derived macrophage phagocytosis and killing assays.

Bone marrow-derived macrophage (BMDM) preparation and the ability to kill A. fumigatus conidia, as determined by assessing CFU, was carried out exactly as described previously (70). The ability of BMDMs to phagocytize A. fumigatus conidia was carried out exactly as described in reference 71. Fresh A. fumigatus conidia were harvested from plates in PBS and filtered through miracloth (Calbiochem). Conidial suspensions were washed three times with PBS and counted using a hemocytometer. For the killing assay, a dilution of 1 × 10^5^ conidia in 200 μl of RPMI 1640 medium with fetal calf serum (FCS) was prepared. For the phagocytosis assay, 1 × 10^6^ conidia were resuspended in 1 ml PBS and inactivated under UV light for 2 h. The percentage of phagocytized conidia was calculated based on conidia cell wall staining with calcofluor white (CFW) (phagocytized conidia are not stained).

### Infection of Galleria mellonella.

Breeding and selection of wax moth larvae, preparation of A. fumigatus conidia, and infection of the last left proleg of larvae with A. fumigatus was carried out exactly as described previously (72).

### Ethics statement.

Eight-week-old gender- and age-matched C57BL/6 mice were bred under the specific-pathogen-free condition and kept at the Life and Health Sciences Research Institute (ICVS) Animal Facility. Animal experimentation was performed following biosafety level 2 (BSL-2) protocols approved by the Institutional Animal Care and Use Committee (IACUC) of the University of Minho, and the ethical and regulatory approvals were given by the Ethics Subcommittee for Life and Health Sciences (074/016). All procedures followed the European Union (EU)-adopted regulations (Directive 2010/63/EU) and were conducted according to the guidelines sanctioned by the Portuguese ethics committee for animal experimentation, Direção-Geral de Alimentação e Veterinária (DGAV).

### Infection of chemotherapeutic mice, fungal burden, and histopathology.

Mice were immunosuppressed intraperitoneally (i.p.) with 200 mg of cyclophosphamide (Sigma) per kg of body weight on days −4, −1, and +2 prior to and postinfection, and subcutaneously with 150 mg/kg hydrocortisone acetate (Acros Organics) on day −1 prior to infection. A. fumigatus conidial suspensions were prepared freshly a day prior to infection and washed three times with PBS. The viability of the administered conidia was determined by growing them in serial dilutions on complete (YAG) medium at 37°C. Mice (*n* = 10/strain) were infected by intranasal instillation of 1 × 10^6^ conidia in 20 μl of PBS. Mice (*n* = 5) which received 20 μl of PBS were used as negative controls. To avoid bacterial infections, the animals were treated with 50 μg/ml of chloramphenicol in drinking water *ad libitum*. Animals were weighed daily and sacrificed if they showed 20% loss in weight, severe ataxia or hypothermia, and other severe complications.

For histological analysis, the lungs were perfused with PBS, excised, and fixed with 10% buffered formalin solution for at least 48 h, and paraffin embedded. Lung sections were stained with hematoxylin and eosin (H&E) for pathological examination. Paraffin-embedded lung tissue sections were also stained for the presence of fungal structures using the Silver Stain kit (Sigma-Aldrich), according to the manufacturer’s instructions. Images were acquired using a BX61 microscope (Olympus) and a DP70 high-resolution camera (Olympus). To quantify lung inflammation of infected animals, inflamed areas on slide images were analyzed using the thresholding tool in ImageJ software (v1.50i, NIH, USA) according to the manufacturer’s instructions.

### Data availability.

The RNA-seq data set can be accessed at NCBI’s Short Read Archive under the Bioproject identifier (ID) PRJNA668271.
